# From Digital Mental Health Interventions to Digital “Addiction”: Where the Two Fields Converge

**DOI:** 10.3389/fpsyt.2019.01017

**Published:** 2020-01-21

**Authors:** Elias Aboujaoude, Lina Gega

**Affiliations:** ^1^ Department of Psychiatry and Behavioral Sciences, Stanford University School of Medicine, Stanford, CA, United States; ^2^ Mental Health and Addiction Research Group, Department of Health Sciences & Hull York Medical School, University of York, York, United Kingdom

**Keywords:** telepsychiatry, telemental health, telemedicine, problematic internet use, internet addiction, privacy

## Abstract

Scientific literature from the last two decades indicates that, when it comes to mental health, technology is presented either as panacea or anathema. This is partly because researchers, too frequently, have planted themselves either in the field of digital mental health interventions (variably called “telepsychiatry”, “digital therapeutics”, “computerized therapy”, etc.), or in that of the problems arising from technology, with little cross-fertilization between the two. Yet, a closer look at the two fields reveals unifying themes that underpin both the advantages and dangers of technology in mental health. This article discusses five such themes. First, the breakneck pace of technology evolution keeps digital mental health interventions updated and creates more potentially problematic activities, leaving researchers perennially behind, so new technologies become outdated by the time they are studied. Second, the freedom of creating and using technologies in a regulatory vacuum has led to proliferation and choice, but also to a Wild-West online environment. Third, technology is an open window to access information, but also to compromise privacy, with serious implications for online psychology and digital mental health interventions. Fourth, weak bonds characterize online interactions, including those between therapists and patients, contributing to high attrition from digital interventions. Finally, economic analyses of technology-enabled care may show good value for money, but often fail to capture the true costs of technology, a fact that is mirrored in other online activities. The article ends with a call for collaborations between two interrelated fields that have been—till now—mutually insular.

## Introduction

In Aesop's ancient fable “Man and Satyr^1^”, the satyr saw the man blow on his hands on a cold winter's day and asked why. The man responded: “so that I can warm them up; can't you see how cold it is?” When they sat down to eat, the man cut a piece of his roast and blew on it before he ate it. The satyr asked why. The man responded: “so that it cools down; can't you see how hot it is?” The satyr, indignantly, walked away. “That's enough,” he said. “I can no longer be your friend since I see you blowing hot and cold with the same breath.” The allegory helps illustrate how digital technology in mental health can be therapeutic and problematic at the same time. This dissonance can cause confusion and mistrust, which leads clinicians, patients and the general public to either disengage from digital mental health interventions, or to underestimate the problems and risks associated with extensive technology use.

As a case in point, a US telemedicine company recently announced positive results in a study of an action video game designed to treat attention deficit and hyperactivity disorder (ADHD), with plans to market it as a prescription digital therapy for ADHD (1). Meanwhile, several studies, including systematic reviews of comorbidity studies [(e.g., (2–4)], have shown a strong link between ADHD and Internet Gaming Disorder (IGD), suggesting that online gaming and other online activities may actually be a causal or contributing factor to developing ADHD. Similarly, a meta-analytic study showed that digital therapies for children and young people's mental health were promising (5), but also fueled anxieties in parents who could not reconcile encouraging their children to use the internet and games for treatment when they were constantly pressing them to reduce screen use, due to concern about the negative impact of digital media on children's emotional and social wellbeing (6, 7).

As this data illustrates, the field of digital mental health interventions is increasingly in collision with that of digital mental health problems and risks. On the one hand, empirical findings into the adverse effects of digital technologies can dissuade adoption of digital mental health interventions such as “serious games”, out of fear that they may fuel a “gaming disorder” (8) or have other negative psychological impact, such as incite violence as result of possible violent content (9, 10), invite bullying by facilitating access to users (11, 12), or encourage social withdrawal (13). On the other hand, overzealous evidence about the efficacy and cost-effectiveness of digital interventions is rarely accompanied by reasonable caution about the uncertainty of positive outcomes with technology, or its risks–financial, legal, ethical and therapeutic—until a brick wall is hit when trying to transfer digital interventions from scholarly ivory towers to real world clinics.

Between the field of digital mental health interventions and that of the risks and adverse effects of digital technologies, rarely has one research group cited or built upon the work of the other (14). Yet reciprocal recognition and collaboration could prove very beneficial: research from both camps over the last two decades suggests shared observations and challenges. In this article, we aim to highlight common themes that have defined research into both the benefits and problems of technology in mental health, and to make recommendations for cross-collaborations between two interrelated fields of research that have traditionally been mutually insular. Rather than review the benefits and harms of technology when it comes to mental health as revealed in research—a very different, much more ambitious endeavor— we instead will focus on how research into both fields has revealed common issues and challenges.

### Speed of Technology: Rapidly Evolving But Easily Outdated

In 1965, Gordon Moore, an engineer and the cofounder of Intel, predicted that the number of transistors that can be fitted on a microchip would double every two years (15). What became known as “Moore's Law” has held up surprisingly well, as has its corollary: that computer processing speed would grow at a similar rate. Digital technology has evolved exponentially, its breakneck speed far outpacing that of mental health research into it.

Slowness is inherent to a robust research process: an investigation question has to be identified, a protocol conceived and approved, participants recruited, an intervention tested, data analyzed, an article written, and peer review performed, before a manuscript can finally be accepted and published. If investigators were testing an antidepressant, there would be little reason to fear the obsoleteness by publication time of the hard earned results. However, when investigating the positive health uses of technology or its negative psychological effects, there is a “moving target” aspect to the process that makes it so that the platform being investigated risks becoming less relevant by the study's end, as users move to ever newer, faster and more engaging platforms. As such, digital technology is, almost by definition, always ahead of the science investigating it, and the research community is perennially “playing catch up”, whether it is weighing in on the merits of the latest digital therapeutic tool being marketed or on the ills of the most recent all-consuming video-game overtaking culture. One result is that clinical researchers often feel ill equipped to address the latest technologies, leaving it to investors, startup executives and engineers to take the lead and steer the field.

### Proliferation of Technology: Freedom But Also a “Wild-West”

The proliferation of digital products means that developers have the freedom to create and distribute diverse technologies, whereas consumers have the freedom to choose and use different products. This “laissez-faire” approach may have been the cornerstone of the classical economic liberalism that propelled innovation and trade in 18^th^ c. Europe. But, in the 21^st^ c. World Wide Web, societies have a duty of care towards technology's end users, especially if these users turn to technology because they seek psychological, social, and emotional support.

Legislative and regulatory bodies have struggled to understand, and respond to, this proliferation of technology. Delayed oversight and lack of consistent and comprehensive protective frameworks have led to negative consequences. In the US, for much of the internet's life, online communications were governed by the 1996 Communications Decency Act, Section 230, which essentially immunized sites from liability for problematic behavior by their users, including bullying, misinformation and sexual predation (16). While Europe has often led the way in internet regulation as a means of protecting consumers, significant safety protections such as the Right To Be Forgotten (17) and the General Data Protection Regulation (18) arrived relatively late (2012 and 2018, respectively). This contributed to a “Wild West”-like online environment that nurtured negative personality traits from narcissism (19, 20) to aggression (20), and may have contributed to the radicalization (20–22), terrorism (20–22) and counter-democratic shifts that have been blamed on the internet (20).

Similarly, despite the field of digital mental health interventions being three decades old (23), it is only in 2017 that, in the US, the Food and Drug Administration initiated a dedicated program for the testing and scrutiny of digital health tools and innovations (24). In the UK, the National Health Service (NHS) introduced a digital, data and technology standards framework as recently as late 2018 (25), forming the basis for the March 2019 Evidence Standards Framework for Digital Health Technologies by the National Institute for Health and Care Excellence (NICE) (25). The legal and regulatory vacuum in which digital mental health interventions have evolved has had deleterious effects on the field's credibility and, one fears, on public health. This is in part because it has allowed to go largely unchallenged false advertising and unfounded scientific claims of the therapeutic value of some “health products” (26). Everyone stands to benefit from comprehensive regulations and legislations that offer broader protection against a digital “Wild West”, without compromising the freedom of using online media and digital mental health interventions.

### Visibility Through Technology: Improved Access But Compromised Privacy

Technology is an open window through which people can access and distribute information, share experiences and communicate with each other. In the past, clinical knowledge was the privilege of a few professionals who communicated it to their patients during face-to-face meetings, if locally available. Technology has improved patient access to specialist knowledge *via* standardized therapy programs, which allow users to learn therapy skills and manage their own care, and *via* remote digital mental health platforms, which allow visits with geographically removed professionals. Technology has also enabled users with mental health problems to share their stories and form peer support networks. Further, it has allowed information exchange between professionals *via* digital media in a way that expedites risk assessment and peer consultations and ensures continuity of care. The open window afforded by technology has come with a price, though: the threat of compromised privacy.

Issues of privacy are at the core of how research into both digital mental health interventions and technology-related problems has evolved. With all too frequent news of hacks into supposedly secure networks, there is growing distrust of digital systems as repositories of health information (27). Besides concerns around electronic medical records, this has meant hesitation on the part of some providers and patients to adopt digital platforms whose confidentiality cannot be guaranteed, even when efficacy data suggests benefit. Legislative actions to try to protect health information in the digital medical record and on telemedicine platforms have, again, lagged behind the increasingly sophisticated modes of violation. As such, regulations, such as the US Health Insurance Portability and Accountability Act (HIPAA) (1996) (28), the Health Information Technology for Economic and Clinical Health Act (HITECH Act) (2009) (29) and the Omnibus Rule (2013) (30), as well as the UK's Data Protection Act (2018) (31), have only been partially successful.

Beyond compromising health information, the post-privacy age ushered in by internet-related technologies has had important effects on psychology. Pre-internet psychological literature delineated several privacy components (32, 33), all of which would seem impacted by our heavily technology-reliant lifestyle (20, 34). Components that, together, constitute privacy include reserve, or the ability to control disclosures; isolation, or the use of geographic distance to separate oneself from others; solitude, or the freedom to place oneself where one cannot be seen or heard; selective intimacy, or the ability to be with an individual or group to the exclusion of others; and anonymity. These privacy components have been shown to mediate psychological functions that are crucial to wellbeing, including contemplation, autonomy, rejuvenation, catharsis, and recovery (33, 34). If the building blocks of privacy are under digital assault, including by facial recognition and Artificial Intelligence (AI) tools now being applied to massive social media databases, then the psychological processes that rely on them may be negatively impacted, with potentially serious consequences (35). As such, privacy violations may be contributing to internet-related psychopathology, just as they threaten the evolution and adoption of digital mental health interventions.

### Attraction of Technology: Strong Pull But Loose Ties

In June 2019, there were 4,422,494,622 internet users, 57.3% of the global population (36). There are over 10,000 mental health-related smartphone apps (37), without counting computerized programs, websites, virtual reality systems and wearables. Designers often approach online users as fickle consumers who are on constant lookout for new digital opportunities—easily drawn in, but just as easily distracted away. Much of the research in online “user experience”, for example, focuses on maximizing “dwell time” (the average time a user spends engaged with a site's content), extending “scroll depth” (how far down the page a user gets when reading content), minimizing “bounce rate” (the percentage of users who navigate away from a site after viewing only one page), and decreasing “time between visits” (38). In Internet psychology, this has been blamed for moving the internet in more extreme directions of representation, including radicalization and narcissism, as desperate page owners vie to attract users at all costs (20).

Like the tenuous commitment of internet users to content, the online definition of a social media “friend” or romantic interest (the app-driven evolution from “dating” to “hooking up” [(20, 39, 40)]) speaks to a similarly “shaky” commitment to relationships developed online. More generally, the virtualization of relationships across digital platforms means looser ties to sites and individuals. This seems true across social media and digital mental health delivery platforms.

The ease of “unfriending” an acquaintance on Facebook or blocking someone on Instagram or Twitter may not differ in fundamental ways from the premature termination of therapy with an e-counselor over a digital mental health portal (41). The provider-patient relationship across many digital platforms is often nonexistent or limited, mirroring digital relationships in the broader sense. The thousands of mental health apps do not appear to have led to measurable population-level mental health benefits. Could it be because, for the most part, there is no trusted provider to recommend or guide their use, or to incorporate them within a more traditional delivery model that encourages patient engagement through supportive accountability?

The attrition problem in digital mental health interventions has been borne out by scientific data emanating from well-designed research studies [(e.g., (42, 43)]. Perhaps because of the lack of a visible, knowable interlocutor on the other side of many online exchanges, engagement with digital experiences tends to be superficial and to lack anchoring. There is ample evidence, though, to suggest that some clinician support is better than none when it comes to outcomes with digital interventions (44–48); in fact, the greater the therapist input, the more effective the interventions seem to be (49, 50). Will fully automated AI platforms that simulate human decision-making and adaptability be able to sustain patients' engagement beyond an initial curiosity-driven stage? Until we find out, we need to invest in human support that strengthens ties and engagement—and ultimately improves outcomes—with digital interventions.

### Economic Value of Technology: Cost-Effective But Costly

There is an assumption that digital mental health interventions offer “good value for money”. By encouraging patient self-management, allowing remote delivery, enabling a less specialist workforce to deliver complex interventions, and reducing waiting lists, digital mental health interventions would be expected to save clinician time and make clinical work more efficient. This assumption comes with several problems. First, we may not be able to forego the traditional intervention for ethical, clinical or practical reasons; e.g., we cannot prohibit patients from seeing their family doctor in favor of following self-management at home. Second, spending for technology is often frontloaded (e.g., cost of software and hardware), whereas savings or improved outcomes are accrued in the longer term, and payers may not have the money to invest upfront. Third, costs may be incurred in one sector and benefits or savings in another, even if their budgets are not linked (e.g., costs for digital therapies are paid by the health clinic or the user, but savings are accrued in the employment sector in the form of less absenteeism). Fourth, the per-patient treatment cost may decrease, but, due to technology's greater reach, the overall number of people treated may go up, thereby increasing total healthcare costs. In the end, the overall economic incentives to adopt digital therapies may be weak, even if they are proven cost-effective.

It is similarly easy to explain away the overuse of internet-related platforms as a means of enhancing one's productivity and, therefore, living standards. “Multi-tasking”, as allowed by a powerful smartphone or by simultaneously opening windows on one's desktop, can feed the illusion that one has cloned himself or developed an extra pair of hands and can now do the work of more than one individual. However, economists still debate whether a “productivity miracle” has been sustained through the successive waves of internet-related technology evolution (51). It turns out that a lot of what people do online—from mindless surfing to online gaming to catching up on celebrity gossip or social media updates—may not necessarily add to their material wealth or the gross domestic product (51). Any benefits from technology-enabled activity have to also be weighed against the costs of treating the population of distracted or otherwise psychologically affected individuals. Whether assessing online distractibility or technology-delivered treatments, there is more to the cost debate than a surface economic reading might suggest.

## Conclusion

Technology blows hot and cold with the same breath when it comes to mental health. Speed, proliferation, visibility, attraction, and economic value are some of the attributes that underlie both its benefits and problems. To our knowledge, no paper has addressed the challenges and themes common to the field of digital mental health interventions and that of the problematic use of digital technology. Research at the intersection of digital technology and mental health has typically focused either on the benefits or the problems, in mutually exclusive fields of enquiry. The resulting literature makes digital technology look like either a panacea or an anathema. The truth, of course, is more complex and more likely to be revealed *via* a “global”, collaborative approach between researchers in the arena of digital mental health interventions and those exploring the risks and negative consequences of technology. In this paper, we made an attempt to bring closer two disparate areas of scholarship. The fact that, as we discussed, similar forces appear to have partially defined both research fields makes such collaborations particularly promising. Joint efforts would empower the research community to understand the psychological, societal, ethical and economic forces at play—and to suggest solutions.

## Author Contributions

Both authors contributed to the conceptualization, researching, and writing of the article.

## Conflict of Interest

The authors declare that the research was conducted in the absence of any commercial or financial relationships that could be construed as a potential conflict of interest.

## Footnotes

1 Satyr: Ancient Greek creature, half-man and half-goat, associated with hedonistic pursuits and the God Dionysus.
